# Distinct Patterns of Gene Gain and Loss: Diverse Evolutionary Modes of NBS-Encoding Genes in Three Solanaceae Crop Species

**DOI:** 10.1534/g3.117.040485

**Published:** 2017-03-28

**Authors:** Lan-Hua Qian, Guang-Can Zhou, Xiao-Qin Sun, Zhao Lei, Yan-Mei Zhang, Jia-Yu Xue, Yue-Yu Hang

**Affiliations:** * Suzhou Polytechnic Institute of Agriculture, 215008, China; † Research Center of Plant Diversity and Systematic Evolution, Institute of Botany, Jiangsu Province and Chinese Academy of Sciences, Nanjing 210014, China

**Keywords:** evolutionary pattern, NBS-encoding genes, phylogenetic relationship, Solanaceae

## Abstract

Plant resistance conferred by nucleotide binding site (NBS)-encoding resistance genes plays a key role in the defense against various pathogens throughout the entire plant life cycle. However, comparative analyses for the systematic evaluation and determination of the evolutionary modes of NBS-encoding genes among Solanaceae species are rare. In this study, 447, 255, and 306 NBS-encoding genes were identified from the genomes of potato, tomato, and pepper, respectively. These genes usually clustered as tandem arrays on chromosomes; few existed as singletons. Phylogenetic analysis indicated that three subclasses [TNLs (TIR-NBS-LRR), CNLs (CC-NBS-LRR), and RNLs (RPW8-NBS-LRR)] each formed a monophyletic clade and were distinguished by unique exon/intron structures and amino acid motif sequences. By comparing phylogenetic and systematic relationships, we inferred that the NBS-encoding genes in the present genomes of potato, tomato, and pepper were derived from 150 CNL, 22 TNL, and 4 RNL ancestral genes, and underwent independent gene loss and duplication events after speciation. The NBS-encoding genes therefore exhibit diverse and dynamic evolutionary patterns in the three Solanaceae species, giving rise to the discrepant gene numbers observed today. Potato shows a “consistent expansion” pattern, tomato exhibits a pattern of “first expansion and then contraction,” and pepper presents a “shrinking” pattern. The earlier expansion of CNLs in the common ancestor led to the dominance of this subclass in gene numbers. However, RNLs remained at low copy numbers due to their specific functions. Along the evolutionary process of NBS-encoding genes in Solanaceae, species-specific tandem duplications contributed the most to gene expansions.

Solanaceae is an extremely diverse family that is distributed in temperate and tropical regions and consists of ∼90 genera and 3000–4000 species (Knapp *et al.* 2004). Many species of this family, including tomato (*Solanum lycopersicum*), potato (*S. tuberosum*), and pepper (*Capsicum annuum*), are valuable crops as well as important model systems for studies of plant development, genetics, and molecular biology (Andolfo *et al.* 2013; Ercolano *et al.* 2012; Knapp *et al.* 2004). However, susceptibility to various pathogens hinders the growth and production of many Solanaceae species, such as tomato *Fusarium* wilt caused by *Fusarium oxysporum*, tomato spotted wilt caused by Tomato spotted wilt virus, potato late blight caused by *Phytophthora infestans*, and pepper bacterial spot disease caused by *Xanthomonas campestris* (Brommonschenkel *et al.* 2000; Tai *et al.* 1999; Vossen *et al.* 2005).

Plants have evolved many effective defense mechanisms, the most important of which is the molecular immune system mediated by disease resistance genes (*R* genes). More than 140 *R* genes have been characterized from different plants. These genes confer resistance to a wide array of pathogens, including bacteria, fungi, oomycetes, viruses, and nematodes (Liu *et al.* 2007; Yang *et al.* 2013). The largest class of *R* genes (∼80%) is the nucleotide binding site (NBS)-encoding genes (Dangl and Jones 2001). An intact NBS-encoding *R* protein structure comprises three principal domains: a toll/interleukin-1 receptor (TIR), coiled-coil (CC), or resistance to powdery mildew8 (RPW8) domain at the N-terminus; an NBS domain in the middle; and an leucine-rich repeat (LRR) domain at the C-terminus (Dangl and Jones 2001; Meyers *et al.* 1999, 2003; Xiao *et al.* 2001). Based on differences in the N-terminal domains, NBS-encoding genes are classified into three subclasses: TIR-NBS-LRR (TNL), CC-NBS-LRR (CNL), and RPW8-NBS-LRR (RNL) (Meyers *et al.* 2003; Shao *et al.* 2014; Zhang *et al.* 2016). The middle NBS domain is highly conserved and encodes several motifs consisting of 10–30 amino acids (aa) (Meyers *et al.* 1999; Yue *et al.* 2012), whereas the C-terminal LRR domain exhibits high diversity and has been associated with pathogen recognition (Dangl and Jones 2001; Kobe and Deisenhofer 1995; Leister and Katagiri 2000).

Evolutionary analyses of NBS-encoding genes at the genome level have been performed in >30 angiosperms (Ameline-Torregrosa *et al.* 2008; Andolfo *et al.* 2014; Bai *et al.* 2002; Chen *et al.* 2010; Guo *et al.* 2011; Jupe *et al.* 2012; J. Li *et al.* 2010; X. Li *et al.* 2010; Lozano *et al.* 2012; Luo *et al.* 2012; Meyers *et al.* 2003; Monosi *et al.* 2004; Mun *et al.* 2009; Porter *et al.* 2009; Tan and Wu 2012; Wan *et al.* 2013; Wei *et al.* 2013; Wu *et al.* 2014; Yang *et al.* 2006, 2008; Zhou *et al.* 2004). Additionally, comparative genomic studies of the evolutionary history of NBS-encoding genes have identified evolutionary characteristics and patterns of NBS-encoding genes in a number of clades. For example, studies targeting *Cucumis sativus*, *C. melo*, and *Citrullus lanatus* of the Cucurbitaceae family have revealed frequent gene losses and a limited number of gene duplications. As a result, the number of NBS-encoding genes in Cucurbitaceae plants is relatively small (<100 genes), especially for *Ci. lanatus*; only 45 NBS-encoding genes have been identified. In addition, although the numbers of NBS-encoding genes in *C. sativus* and *C. melo* are similar, they are a result of different gene duplication and loss events (Lin *et al.* 2013). A comparative genomic analysis of four Poaceae species revealed that the number of NBS-encoding genes in *Zea mays* was only half that in *Sorghum bicolor* and *Brachypodium distachyon* and a fourth of that in *Oryza sativa*. One possible reason is that transposable elements caused massive pseudogenization of the NBS-encoding genes followed by gene losses in *Z. mays* (J. Li *et al.* 2010). The evolutionary patterns of NBS-encoding genes have also been studied in other plants. For example, both Fabaceae and Rosaceae show a consistent expanding pattern (Jia *et al.* 2015; Shao *et al.* 2014), whereas Brassicaceae species exhibit a pattern of expansion followed by contraction (Zhang *et al.* 2016).

High-quality genome sequences of tomato, potato, and pepper in Solanaceae are available. Many functional *R* genes have also been cloned from Solanaceae species. For example, *Rpi-blb2* of potato provides *P. infestans* resistance (Vossen *et al.* 2005), *SW5* of tomato confers tomato spotted wilt virus resistance (Brommonschenkel *et al.* 2000), and *BS2* of pepper protects against *X. campestris* (Tai *et al.* 1999). The evolutionary characteristics of certain NBS-encoding genes in the tomato and potato genomes have also been analyzed (Andolfo *et al.* 2013, 2014; Jupe *et al.* 2012; Lozano *et al.* 2012; Xu *et al.* 2011). A systematic evaluation of NBS-encoding genes at the genome level in more Solanaceae species is required to obtain a better understanding of the resistance to the diversity of pathogen invasions. Recently, Wei *et al.* (2016) performed a comprehensive study of Solanaceae species and observed dramatic *R* gene number variation caused mainly by a few loci. These findings suggest a need for further exploration of the causes of these dramatic variations and elucidation of the gene loss/gain events in detail. Using the whole-genome sequence data from three Solanaceae species (tomato, potato, and pepper), we aim to unravel the evolutionary features and patterns of NBS-encoding genes and further investigate the mechanisms underlying evolutionary changes.

## Materials and Methods

### Identification and classification of NBS-encoding genes

**Figure 1:**
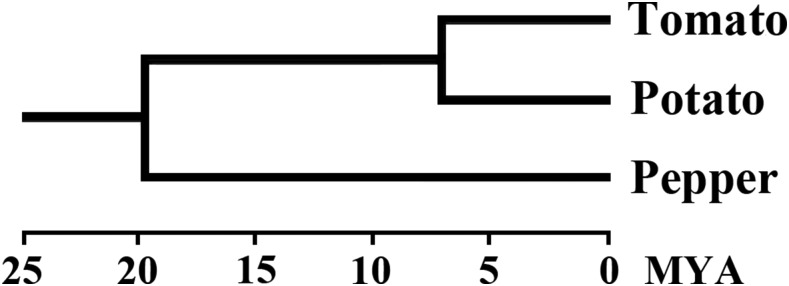
Phylogenetic relationship of tomato, potato, and pepper. Times of divergence (million years ago, MYA) are from Wu and Tanksley (2010), Tu *et al.* (2010), and Wang *et al.* (2008).

The whole genomes of three Solanaceae species, tomato (*S. lycopersicum*), potato (*S. tuberosum*), and pepper (*C. annuum*) (Figure 1), were used in the present study. Genomic sequences of tomato and potato were downloaded from the Phytozome database (http://www.phytozome.org/; PhytozomeV9). The genomic sequences of pepper (cultivated *C. annuum* accession Zunla-1_v2.0) were obtained from the Pepper Genome Database (http://peppersequence.genomics.cn/page/species/index.jsp). A process including two steps was then used to identify candidate NBS-encoding genes (Shao *et al.* 2015). First, BLAST and hidden Markov model (HMM) searches using the NB-ARC domain (Pfam accession number: PF00931) as the query sequence were simultaneously performed to scan and identify the candidate NBS-encoding genes in the genomes of tomato, potato, and pepper. For the BLAST method, the threshold expectation value was set to 1.0 as described in a previous study (J. Li *et al.* 2010). The default parameter settings were used for the HMM search (http://hmmer.org). Second, all obtained sequence hits using BLAST or HMM searches were merged together and the redundant hits were removed. The remaining sequences were subjected to online Pfam analysis (http://pfam.sanger.ac.uk/) to further confirm the presence of the NBS domain by an *E*-value of 10^−4^. All identified NBS-encoding genes were analyzed using the Pfam database (http://pfam.janelia.org/), SMART protein motif analyses (http://smart.embl-heidelberg.de/), and Multiple Expectation Maximization for Motif Elicitation (MEME) to determine if they encoded TIR, RPW8, or LRR motifs. The CC motifs were detected by the COILS program (http://www.ch.embnet.org/software/COILS_form.html) (Lupas *et al.* 1991) with a threshold of 0.9 followed by visual inspection.

### Chromosomal distribution of NBS-encoding genes and cluster assignment

In each Solanaceae genome, the chromosomal locations of all identified NBS-encoding genes were determined by retrieving relevant information from the downloaded annotation data. We then examined the numbers of CNL, TNL, and RNL subclass NBS-encoding genes on different chromosomes. The criterion of gene cluster assignment followed the protocol used for *Medicago truncatula* (Ameline-Torregrosa *et al.* 2008): if two neighboring NBS-encoding genes were located within 250 kb on a chromosome, the two genes were regarded as members of the same gene cluster. Based on this criterion, the NBS-encoding genes in each Solanaceae genome were assigned to a number of singleton loci and clustered loci, which were mapped along the chromosomes.

### Sequence alignment and conserved motif analysis of NBS domain

The amino acid sequences of the NBS domain were extracted from the identified NBS-encoding genes and used for multiple alignments using ClustalW integrated in MEGA 5.0 with default parameter settings (Tamura *et al.* 2011). NBS domain-encoding sequences that were too short [shorter than two-thirds of a regular NBS domain (∼290 aa) or too divergent (the genes whose NBS domains could not be well aligned with others, and the aligned lengths are shorter than two-thirds of a regular NBS domain] were removed to prevent them from interfering with the alignment and subsequent phylogenetic analysis. The resulting amino acid sequence alignments were then manually corrected in MEGA 5.0 and used to guide the alignments of nucleotide sequences. Conserved protein motifs were analyzed by online MEME (Bailey *et al.* 2006) and WebLogo (Crooks *et al.* 2004) with default parameter settings. In addition, structural motif annotation was performed using the online Pfam and SMART tools.

### Phylogenetic and gene loss/duplication analysis of NBS-encoding genes in the tomato, potato, and pepper genomes

To explore the relationships of NBS-encoding genes in the tomato, potato, and pepper genomes, a phylogenetic tree was reconstructed based on the nucleotide sequences of the conserved NBS domains. The nucleotide sequences were aligned as described above. Phylogenetic trees were reconstructed using the maximum likelihood method based on a GTR model, and the reliability of the internal nodes of the tree was assessed by calculating the SH-aLRT branch support (Guindon *et al.* 2010). In addition, gene loss/duplication events during the speciation of tomato, potato, and pepper were restored by reconciling the NBS-encoding gene phylogenetic tree with the real species tree using Notung software (Stolzer *et al.* 2012).

### Synteny analyses within and across Solanaceae genomes

Synteny analysis was performed using the MCScanX package (http://chibba.agtec.uga.edu/duplication/) to identify syntenic blocks within a genome or between different genomes through BLASTp searches. The purposes of synteny analysis were to explore the pattern of conservation of NBS-encoding gene loci among the three Solanaceae genomes and to identify NBS-encoding gene pairs resulting from segmental duplications within a Solanaceae genome.

### NBS-encoding gene duplication analysis

There are three types of NBS-encoding gene duplications: local tandem duplication, ectopic duplication, and segmental duplication (Leister 2004). The closely related NBS-encoding genes were checked clade by clade on the reconstructed NBS-encoding gene phylogeny. Their chromosomal locations were used in combination with their within-genome syntenic relationships to estimate the number of duplicated genes resulting from each type of duplication. To increase the accuracy of the estimation, we only considered duplications occurring on terminal branches leading to the three Solanaceae species because accumulated chromosomal activities (splits, fusions, and rearrangements) are difficult to distinguish in ancient tandem, ectopic, and segmental duplications.

### Data availability

All the identified NBS-encoding genes and their alignments can be made available upon request. The authors state that all data necessary for confirming the conclusions presented in the article are represented fully within the article.

## Results

### Identification and classification of NBS-encoding genes in three Solanaceae genomes

BLAST and HMM searches identified 255, 447, and 306 NBS-encoding genes from the genomes of tomato, potato, and pepper, respectively (Table 1). The number of NBS-encoding genes in the potato genome was largest and was 1.75 and 1.46 times greater than those in the tomato and pepper genomes, respectively. All three subclasses of NBS-encoding genes, TNLs, CNLs, and RNLs, were identified from the three Solanaceae species based on their domain composition and primary phylogeny analysis. Among the genes, CNLs accounted for the overwhelming majority, with proportions of 87.0, 83.7, and 94.1% in tomato, potato, and pepper, respectively. TNLs occupied 12.2, 15.6, and 5.2% in the corresponding species, whereas the numbers of RNL genes were smallest, with only two or three genes in each species. As shown in Table 1, not all identified genes had intact structures for all three domains (N-terminal-NBS-LRR). There were 109, 126, and 71 intact NBS-encoding genes present in the tomato, potato, and pepper genomes, respectively, accounting for only 42.7, 28.2, and 23.2% of the total numbers.

**Table 1:** The number of identified NBS-encoding genes in the three Solanaceae genomes

Domain Compositions	Tomato	Potato	Pepper
TNL subclass	31 (12.2%)	70 (15.6%)	16 (5.2%)
* TNL* (Intact)	17	31	5
* TN*	7	18	0
* NL*	3	4	4
* N*	4	17	7
CNL subclass	222 (87.0%)	374 (83.7%)	288 (94.1%)
* CNL* (Intact)	90	93	65
* CN*	6	72	29
* NL*	50	129	92
* N*	76	80	102
RNL subclass	2 (0.8%)	3 (0.7%)	2 (0.7%)
* RNL* (Intact)	2	2	1
* RN*	0	0	0
* NL*	0	0	1
* N*	0	1	0
Total number	255	447	306

TNL, TIR-NBS-LRR; CNL, CC-NBS-LRR; RNL, RPW8-NBS-LRR.

### Distribution and organization of NBS-encoding genes in Solanaceae genomes

Although all chromosomes contain NBS-encoding genes, these genes were unevenly distributed among different chromosomes (Supplemental Material, Figure S1). For example, Chr (chromosome) 4 of potato and tomato contained the most genes (69 and 51 genes, respectively) in each species, whereas Chr 3 of potato and tomato contained the fewest genes (five and seven genes, respectively). In contrast to potato and tomato, Chr 3 and 6 of pepper contained the most and fewest genes (43 and 4 genes), respectively.

Uneven distributions were also observed among different subclasses of NBS-encoding genes. Chr 4 of potato and tomato and Chr 3 of pepper contained the most CNLs, whereas Chr 1 of potato and tomato and Chr 12 of pepper contained the most TNLs; all chromosomes contained CNLs, but two chromosomes of potato and tomato each and seven chromosomes of pepper did not contain TNLs. There were too few RNLs for this analysis. The majority of NBS-encoding genes were organized into clusters rather than singletons in Solanaceae, and their ratios ranged from 1.95 to 4.59 among the three genomes (Table 2). Potato contained the most clustered loci and genes, and its clustered loci contained the most genes (4.65 genes/locus) among the three species on average.

**Table 2:** Organization of NBS-encoding genes in the three Solanaceae genomes

Loci and Genes	Tomato	Potato	Pepper
No. of chromosome-anchored loci (and genes)	129 (253)	159 (447)	138 (260)
No. of singleton loci (no. of genes)	79 (79)	80 (80)	88 (88)
No. of clustered loci (no. of genes)	50 (174)	79 (367)	50 (172)
Clustered genes/singleton genes	2.2	4.59	1.95
Average no. of genes in clusters	3.48	4.65	3.44
No. of clusters with 10 or more genes	1	10	1
No. of genes in the largest cluster	13 (Chr 4)	22 (Chr 4)	13 (Chr 3)

No., number; Chr, chromosome.

**Figure 2:**
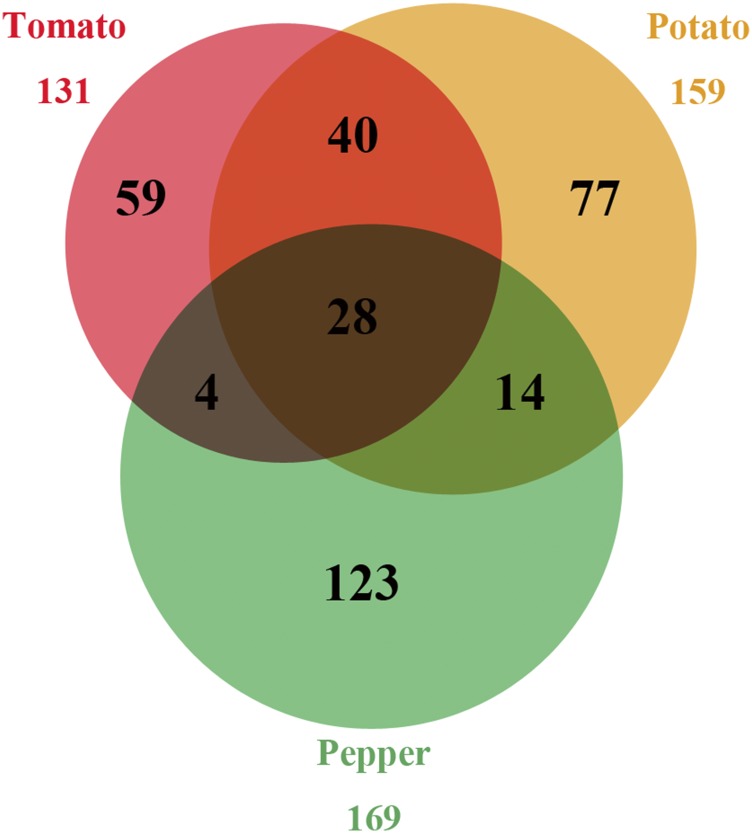
NBS loci in the tomato, potato, and pepper genomes. NBS, nucleotide binding site.

Our survey identified a total of 159, 131, and 169 NBS loci assigned to the genomes of potato, tomato, and pepper, respectively (Figure 2 and Figure S1), whereas syntenic analysis revealed that only 28 loci were maintained at collinear positions in all three genomes, 58 loci (tomato and potato: 40; tomato and pepper: 4; and potato and pepper: 14) were preserved in only two genomes, and 259 loci were species-specific (Figure 2). These distribution patterns suggest that some NBS-encoding genes (syntenic) were inherited from a common ancestor, whereas others (species-specific) arose after the species diverged.

### Characterization of class-specific signatures among three NBS-encoding gene classes

**Figure 3:**
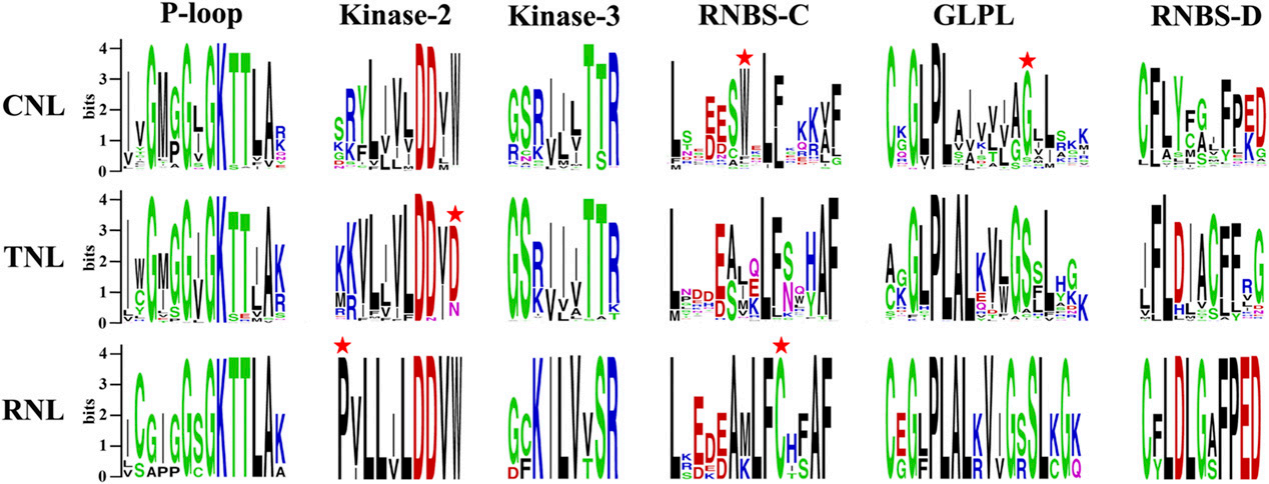
Six conserved motifs in NBS domains of the three Solanaceae species. The amino acids of the six motifs were extracted. Different conserved amino acids among TNL, CNL, and RNL subclass genes are labeled with a red star. The details of the amino acids of the whole NBS domain of NBS-encoding genes in the three Solanaceae species are shown in Figure S2. CNL, CC-NBS-LRR; NBS, nucleotide binding site; RNL, RPW8-NBS-LRR; TNL, TIR-NBS-LRR.

To explore the structural components and confirm the homology of all NBS-encoding genes from the three Solanaceae species, the conserved motifs within the NBS domain were identified by MEME (Bailey *et al.* 2006) and WebLogo (Crooks *et al.* 2004). A total of six conserved motifs were identified in the NBS domains of the three Solanaceae species. From the N-terminus to the C-terminus, these domains are the P-loop, Kinase-2, Kinase-3, RNBS-C, GLPL, and RNBS-D (Figure 3). The first five motifs exhibited high similarity among all three subclasses of NBS-encoding genes, suggesting homology of all NBS domains. Although the RNBS-D motif varied significantly among the three subclasses, it was highly conserved within each subclass. Further analysis revealed that a few amino acids in the conserved motifs could be used as preliminary labels to identify CNL, TNL, or RNL subclass genes, such as tryptophan (W) at the seventh position of RNBS-C and aspartic acid (D) at the 13th position of GLPL in CNL genes; aspartic acid (D) at the final position of Kinase-2 in TNL genes; and proline (P) at the first position of Kinase-2 and cysteine (C) at the 10th position of RNBS-C in RNL genes (Figure 3). Therefore, the subclass of an NBS-encoding *R* gene could be determined by the characteristics of the motif sequences.

**Figure 4:**
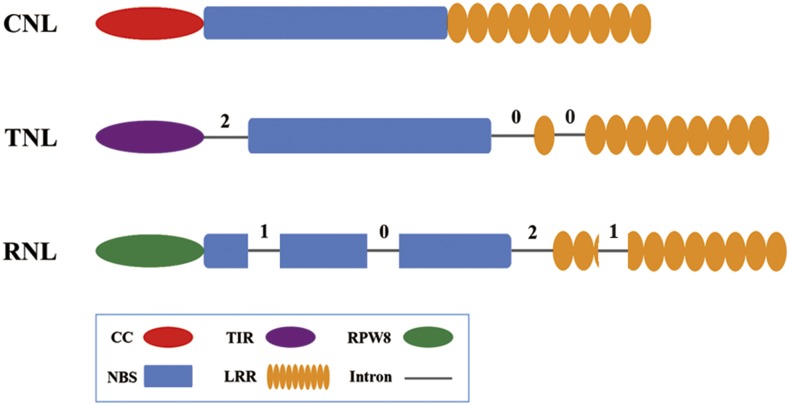
Exon/intron structures among the CNL, TNL, and RNL subclasses. CNL, CC-NBS-LRR; NBS, nucleotide binding site; RNL, RPW8-NBS-LRR; TNL, TIR-NBS-LRR.

Our previous study revealed distinct intron positions and phases among the classes of NBS-encoding genes in bryophytes (Xue *et al.* 2012). In Solanaceae, three introns with conserved positions and phases are shared by most TNL genes (Figure 4), with the first intron separating the TIR domain and the NBS domain, the second intron separating the NBS domain and the whole LRR domain, and the third intron separating the first and remaining LRR domains. Angiosperm RNLs also share four class-specific introns, two inside the NBS domain and two inside the LRR domain. CNL genes do not have shared introns. Although some CNL genes have introns, these are more likely to have been gained by specific genes. This finding is in agreement with the intron/exon structure of NSB-encoding genes in bryophytes and other angiosperm families (Xue *et al.* 2012; Shao *et al.* 2014; Zhang *et al.* 2016).

### Phylogenetic analysis of NBS-encoding genes in three Solanaceae species

**Figure 5:**
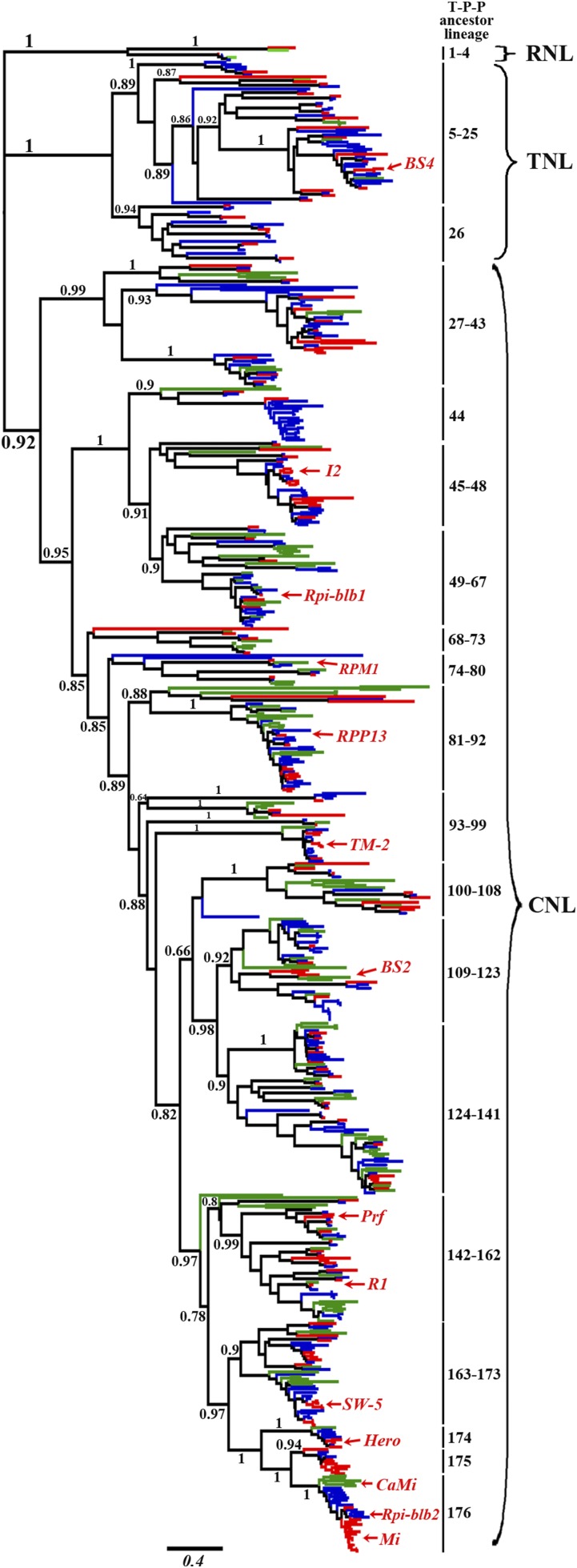
Phylogenetic relationships of NBS-encoding genes in tomato, potato, and pepper based on conserved NBS domains. The red, blue, and green lines represent NBS-encoding genes in tomato, potato, and pepper, respectively. NBS-encoding gene subclasses (CNL, TNL, and RNL) and support values >0.75 (SH-aLRT values) for basal nodes are shown. The T-P-P ancestor lineage indicates the NBS-encoding genes from the common ancestor of tomato, potato, and pepper (a total of 176). Homologous genes with known-function disease resistance genes in the tomato, potato, and pepper genomes obtained by BLASTp homology comparison are labeled with red arrows and the names of functional genes (*BS4*, *I2*, *Rpi-blb1*, *RPM1*, *RPP13*, *TM-2*, *BS2*, *Prf*, *R1*, *SW-5*, *Hero*, *CaMi*, *Rpi-blb2*, and *Mi-1*). The detailed information on the phylogenetic tree of NBS-encoding genes identified from tomato, potato, and pepper, including gene names, evolutionary relationships among genes, and supporting values of all nodes, are shown in Figure S3. CNL, CC-NBS-LRR; NBS, nucleotide binding site; RNL, RPW8-NBS-LRR; TNL, TIR-NBS-LRR; T-P-P, tomato, potato, and pepper.

To determine the phylogenetic relationship of NBS-encoding genes and to uncover their evolutionary history in Solanaceae, phylogenetic analysis was performed using the nucleotide sequences of the NBS domains. To exclude interference from “noisy characters,” too short or extremely divergent NBS domains were removed from the alignment and subsequent phylogenetic analysis. Collectively, 603 genes (tomato: 163; potato: 310, and pepper: 130) were aligned and used to reconstruct the evolutionary history of NBS-encoding genes. The maximum likelihood phylogenetic tree was composed of three monophyletic clades with support values over 0.9, and the majority of internal nodes received strong (>0.8) support values (Figure 5). The phylogeny constructed using all identified NBS domains exhibited poor robustness reflected by more branches with support values <0.5 as well as more long branches, because the too short sequences maintained too limited phylogenetic signals causing stochastic errors and too divergent sequences gave fake signals causing systematic errors (Figure S4).

The three clades exactly represent the divergence of RNLs, TNLs, and CNLs, and all genes fall into groups of corresponding classes without exception. The retrospective analysis suggested that the six genes in the RNL clade are descendants of four ancestral genes (T-P-P ancestor lineage 1–4) in the common ancestor of tomato, potato, and pepper. The TNL clade consists of two sister subclades; one subclade is derived from 21 ancestral genes (T-P-P ancestor lineage 5–25), whereas the other subclade is derived from only one ancestral gene (T-P-P ancestor lineage 26). The bacterial spot disease resistance gene *BS4* cloned from tomato is a TNL gene and is the only functionally characterized TNL gene in Solanaceae (Figure 5). The CNL clade is the largest branch in this phylogeny; it consists of 514 CNL genes derived from 150 ancestral genes (T-P-P ancestor lineage 27–176) and contains many known-function *R* genes.

### Dynamic patterns of NBS-encoding genes through evolutionary history

**Figure 6:**
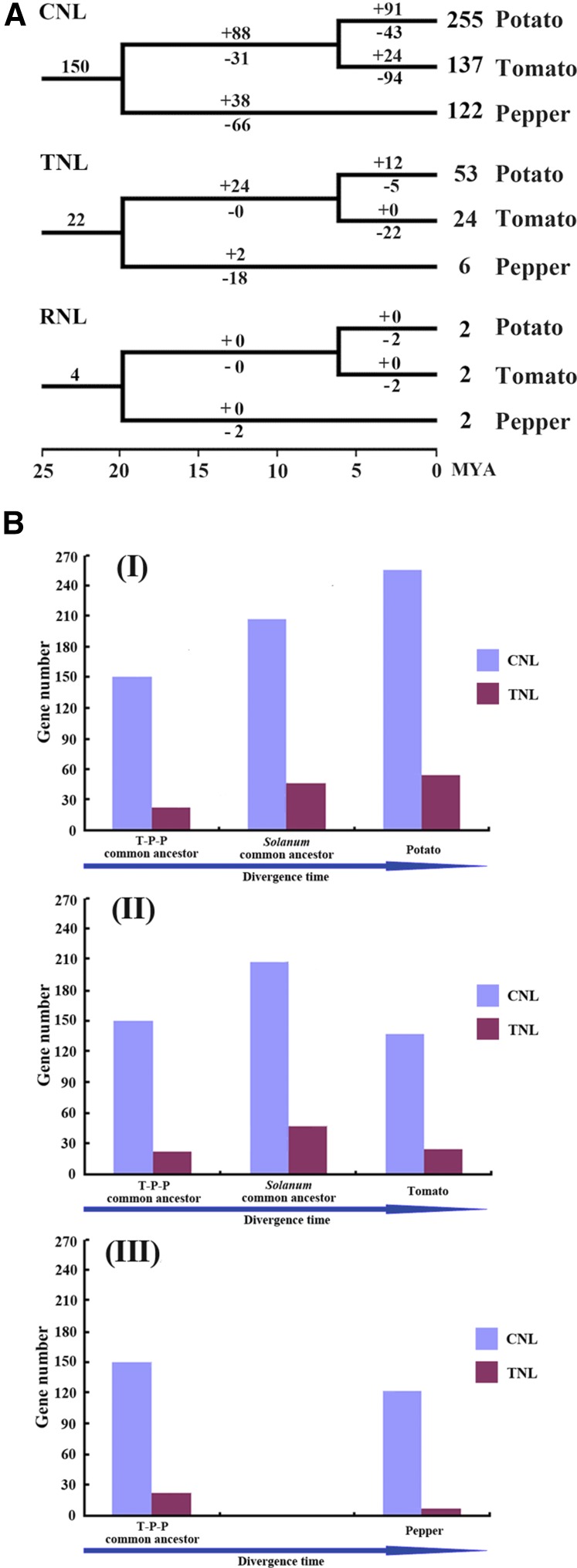
Dynamic patterns of NBS-encoding genes in the three Solanaceae genomes. (A) Loss/duplication events of NBS-encoding genes during the speciation of tomato, potato, and pepper. Gene losses and duplications are indicated by numbers with “–”or “+” on each branch. Dates of divergence of tomato, potato, and pepper are shown as MYA (Wang *et al.* 2008; Tu *et al.* 2010; Wu and Tanksley 2010). Detailed information for gain and loss of NBS-encoding genes is shown in Figure S5. In addition, the detailed information for gain and loss, and the loss/duplication events of NBS-encoding genes based on the phylogeny of NBS domains of all identified NBS-encoding genes are shown in Figure S6 and Figure S7, respectively. (B) Evolutionary modes of NBS-encoding genes in the three Solanaceae species. (I) The gene number variation from the T-P-P ancestor to potato. (II) The gene number variation from the T-P-P ancestor to tomato. (III) The gene number variation from the T-P-P ancestor to pepper. CNL, CC-NBS-LRR; MYA, millions of years ago; NBS, nucleotide binding site; RNL, RPW8-NBS-LRR; TNL, TIR-NBS-LRR; T-P-P, tomato, potato, and pepper.

To investigate the evolutionary patterns of NBS-encoding genes in the three Solanaceae species, the phylogenetic tree based on the conserved NBS domain sequences was reconciled with the real species tree to restore gene loss and duplication events that occurred during the speciation of the three species. The retrospective analysis revealed 22 TNL, 150 CNL, and 4 RNL genes in the common ancestor of tomato, potato, and pepper. However, these ancestral genes experienced differential evolutionary patterns in these three Solanaceae species. Species-specific gene duplication and loss events (Figure 6A) reflect diverse evolutionary patterns of the NBS-encoding genes in Solanaceae. The details of the NBS-encoding gene duplications and losses involved in the process of formation of tomato, potato, and pepper species are shown in Figure S5.

Pepper, which diverged first, lost 86 genes (18 TNLs, 66 CNLs, and 2 RNLs) and gained 40 genes by duplication (2 TNLs and 38 CNLs). The ratio of gene loss to gain was >2. The common ancestor of tomato and potato, after splitting from the pepper lineage, lost 31 genes and gained 112 genes (24 TNLs and 88 CNLs), resulting in an increase in the total gene number. However, tomato and potato exhibited different patterns after their split. Tomato gained 24 genes (all CNLs) but lost 118 genes (22 TNLs, 94 CNLs, and 2 RNLs); as a result, the total number of NBS-encoding genes in the tomato genome decreased. Potato gained 103 genes (12 TNLs and 91 CNLs) and lost only 50 genes (5 TNLs, 43 CNLs, and 2 RNLs), and thus, the gene number continued to expand in the potato genome after the split of the two *Solanum* species. Thus, in the long-term, the NBS-encoding genes in the three Solanaceae species exhibited dynamic and distinct patterns at different historical periods and in different species: potato showed a “consistent expansion” pattern, tomato exhibited a pattern of “first expansion and then contraction,” and pepper presented a “shrinking” pattern, based on the final outcome (Figure 6B).

## Discussion

### The species specificity of the number of NBS-encoding genes

The number of NBS-encoding genes varies greatly among plant lineages/species, even between closely related species (Jacob *et al.* 2013). For example, most legumes have two to three times more NBS-encoding genes than Brassicaceae plants (Shao *et al.* 2014; Zhang *et al.* 2016; Arakaki *et al.* 2011). The differences are greater than twofold between the Poaceae plants maize (*Z. mays*) and *So. bicolor*, and are nearly fourfold between rice and maize (J. Li *et al.* 2010; Arakaki *et al.* 2011; Yamane *et al.* 2006). In our study, the gene number discrepancy among the three Solanaceae species was also quite large, with 255, 447, and 306 in tomato, potato, and pepper, respectively. The numbers of NBS-encoding genes identified from tomato, potato, and pepper were generally consistent with previous studies (Andolfo *et al.* 2013, 2014; Jupe *et al.* 2012; Shao *et al.* 2016; Xu *et al.* 2011). However, we identified fewer genes compared with Wei *et al.* (2016) because we excluded genes lacking the NBS domain, whereas Wei *et al.* (2016) included these partial genes with only TIR or LRR domains present. Those partial genes may not be the remnants of NBS genes. For example, the Receptor-like Kinase and Receptor-like Transmembrane Protein genes, which belong to other gene families, also contain LRR domains (Dangl and Jones 2001).

We were able to infer not only the numbers of duplication and loss events since the common ancestor of the three Solanaceae species (Figure 6A), but also each relevant gene loss/gain event (Figure S5). Our analysis revealed a large number of species-specific duplication and loss events in Solanaceae. These independent events gave rise to the differences in gene numbers among species. We concluded that potato showed a “continuous expansion” pattern by gaining more genes than were lost at two stages (before and after the split of tomato), and that the tomato NBS-encoding genes exhibited an “expansion followed by contraction” pattern because tomato gained more genes before the split of potato but subsequently lost more genes. Although the status of the middle nodes in the evolution of pepper cannot be evaluated, its overall pattern is a “shrinking” pattern. Therefore, these distinct patterns of gene gain and loss resulted in differences in the number of NBS-encoding genes among the three species. Furthermore, the same subclass of NBS-encoding genes also exhibited different patterns among the three species. The patterns of TNLs and CNLs were similar to that of NBS-encoding genes overall in the corresponding species. By contrast, the number of RNL genes was too small and thus had little effect on the overall changes in the NBS-encoding genes. Furthermore, surveying the types of each gene duplication event revealed that tandem duplication was responsible for the majority (60.8, 60.0, and 47.5% for potato, tomato, and pepper, respectively) of gene expansion events, whereas ectopic duplication contributed 39.2, 38.6, and 40% of new NBS-encoding genes in potato, tomato, and pepper, respectively (Table 3).

**Table 3:** Contributions of three duplication types in producing new NBS-encoding genes during the evolution of the three Solanaceae species

Different Types of Duplications	Tomato	Potato	Pepper
Total no. of new duplicated genes	70	194	40
Local tandem duplication	42	118	19
Ectopic duplication	27	76	16
Segmental duplication	0	0	1
Unanchored genes	1	0	4

No., number.

### The numbers of CNL and TNL genes and the causes

The numbers of CNLs in the tomato, potato, and pepper genomes were significantly higher than those of other subclasses, accounting for 87, 83.7, and 94.1% of the NBS-encoding genes in each species, respectively. By recovering the NBS-encoding genes in the common ancestral genome of these three species, a total of 150 CNL ancestral genes, 22 TNL ancestral genes, and 4 RNL ancestral genes were reconciled. Although NBS-encoding genes have undergone different degrees of gene duplication and loss after the split of the three species, the earlier expansion in their common ancestor ensured the maintenance of the dominance of CNLs in gene number.

A greater number of CNLs is a common phenomenon among angiosperms. For example, CNLs are the majority among the legume species soybean (*Glycine max*), common bean (*Phaseolus vulgaris*) and pigeon bean (*Cajanus cajan*) (Shao *et al.* 2014); in melon (*C. melo*), watermelon (*Ci. lanatus*), and cucumber (*C. sativus*) in Cucurbitaceae (Jia *et al.* 2015); poplar (*Populus trichocarpa*) in Salicaceae (Yang *et al.* 2008); and peach (*Prunus persica*) and strawberry (*Fragaria vesca*) in Rosaceae (Jia *et al.* 2015). As an extreme case, monocots have only CNL genes due to the loss of their TNL genes near dicot/monocot differentiation (J. Li *et al.* 2010). We previously found that CNLs began to expand earlier than TNLs during angiosperm evolution (Shao *et al.* 2016). When the common ancestor of the three Solanaceae species emerged, its genome possessed many more CNLs than TNLs or RNLs, and thus, the predominance of CNLs in Solanaceae was inborn. CNLs are thought to possess much greater sequence diversity than TNLs, which helped broaden their resistance spectrum. By searching all functional NBS-encoding genes we found that, among 110 characterized genes, >90% belong to the CNL subclass, which confers resistance to diverse pathogens, including fungi, bacteria, protists, and viruses (Liu *et al.* 2007; Yang *et al.* 2013). This distribution may indicate that CNLs have broader resistance than TNLs and thus could represent a larger pool of tools for recognizing multiple pathogen effectors. Notably, the ratio of CNL genes to TNL genes was highest in the pepper genome, with a value of 18 (Table 1). To date, the five known functional NBS-encoding *R* genes identified from pepper are all CNL subclass genes: *Bs2* and *CaMi* confer resistance to strains of *X. campestris* pv. *vesicatoria* and root-knot nematodes (Chen *et al.* 2007; Tai *et al.* 1999), respectively; *Pvr4* provides extreme resistance to a broad range of potyviruses (Dogimont *et al.* 1996); *Tsw* controls the hypersensitive response to most tomato spotted wilt virus isolates (Boiteux 1995); and *L* confers resistance to *Tobamovirus* spp. (Tomita *et al.* 2011). Therefore, we speculate that pepper CNLs expanded because of their diverse resistance in the whole life cycle.

The analysis of the chromosomal distribution of NBS-encoding genes indicated that the distributions of CNL and TNL genes among different chromosomes are uneven (Figure S1). An uneven distribution was also observed in several legume and Brassicaceae species (Shao *et al.* 2014; Zhang *et al.* 2016). Our surveys showed that tandem duplication and ectopic duplication were the main contributors to gene expansion events (Table 3). Random ectopic gene duplications and gene loss likely shaped the uneven distributions of CNL and TNL genes on different chromosomes, and this difference is made more apparent through local tandem duplications. Plants developed a strategy of generating a series of alleles and forming gene clusters by tandem duplication to overcome the limitations of *R* gene diversity under divergent selection during the coevolution of plants and pathogens (Le Roux *et al.* 2015; Michelmore and Meyers 1998).

### The numbers of RNL genes and their functional characteristics

RNLs in Solanaceae were classified into CNLs in previous studies (Andolfo *et al.* 2013, 2014; Jupe *et al.* 2012; Lozano *et al.* 2012; Xu *et al.* 2011) due to the extremely small number of RNLs in Solanaceae compared with the two other subclasses. RNLs are limited among all angiosperms (Collier *et al.* 2011; Shao *et al.* 2014; Zhang *et al.* 2016). For example, in Poaceae, some species, such as *O. sativa* and *So. bicolor*, have only one RNL gene (Shao *et al.* 2016). Although RNLs were initially classified into the CNL subclass, RNLs feature unique N-terminal domains, characteristic exon/intron structure, and overall sequence conservation (Meyers *et al.* 2003). A subsequent phylogenetic analysis revealed that this class of NBS-encoding genes is a sister group to CNLs and confirmed that they should be evolutionarily equivalent to CNLs; thus, this subclass was named RNL (Shao *et al.* 2014). *N-required gene* 1 (*NRG1*) of *Nicotiana benthamiana* and *Activated Disease Resistance 1* (*ADR1*) of *Arabidopsis thaliana* (Collier *et al.* 2011) are the only two functionally characterized RNL genes. However, these genes do not participate directly in pathogen recognition but instead act as “helpers” to other pathogen recognition NBS-encoding *R* genes (TNL and CNL genes) and play roles in the downstream signaling pathways of antidisease responses. Although counter to the traditional understanding of the NBS-encoding genes, RNLs are important constituents of disease resistance pathways in plants and are required for basic defense responses (Bonardi *et al.* 2011; Collier *et al.* 2011). Therefore, we speculate that, because the function of RNLs is relatively simple, this subclass of genes does not require violent expansions like CNLs and TNLs, which respond to diverse and rapidly evolving pathogens; only a small number of RNLs is required to minimize the fitness cost.

In conclusion, genome-wide comparative analyses of NBS-encoding resistance genes in three Solanaceae species, potato, tomato, and pepper, were performed from multiple aspects. Phylogenetic analysis revealed three monophyletic groups in accordance with the classification of TNLs, CNLs, and RNLs. A total of 176 ancestral genes were reconciled in the common ancestor of the three Solanaceae species, and current genes should be derived from these ancestral genes. The analysis of gene loss/duplication events indicated species-specific evolutionary modes of NBS-encoding genes in Solanaceae, possibly to cope with different pathogens. The expansion of CNLs in the common ancestor is likely responsible for the present higher number of CNL genes in Solanaceae compared with TNL and RNL genes. Overall, this study elucidated the evolution of NBS-encoding genes in Solanaceae and could assist future functional characterization studies.

## Supplementary Material

Supplemental material is available online at www.g3journal.org/lookup/suppl/doi:10.1534/g3.117.040485/-/DC1.
