# Supplementary figures and images for: Distinct Patterns of Gene Gain and Loss: Diverse Evolutionary Modes of NBS-Encoding Genes in Three Solanaceae Crop Species
Source: G3 (Bethesda). 2017 Mar 28;7(5):1577–85. doi: 10.1534/g3.117.040485 (PMC5427506; doi:10.1534/g3.117.040485)

**A**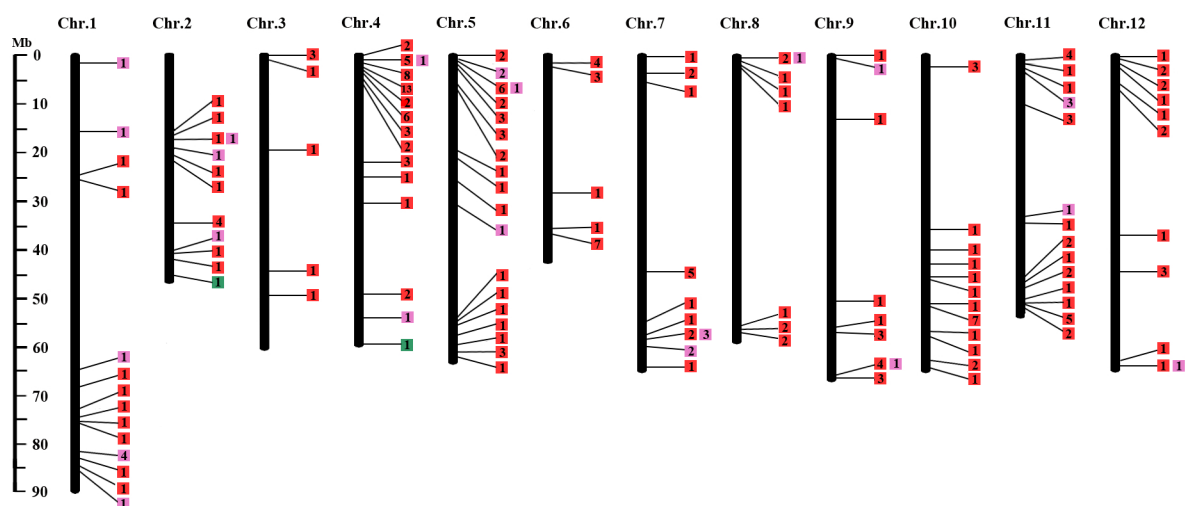**B**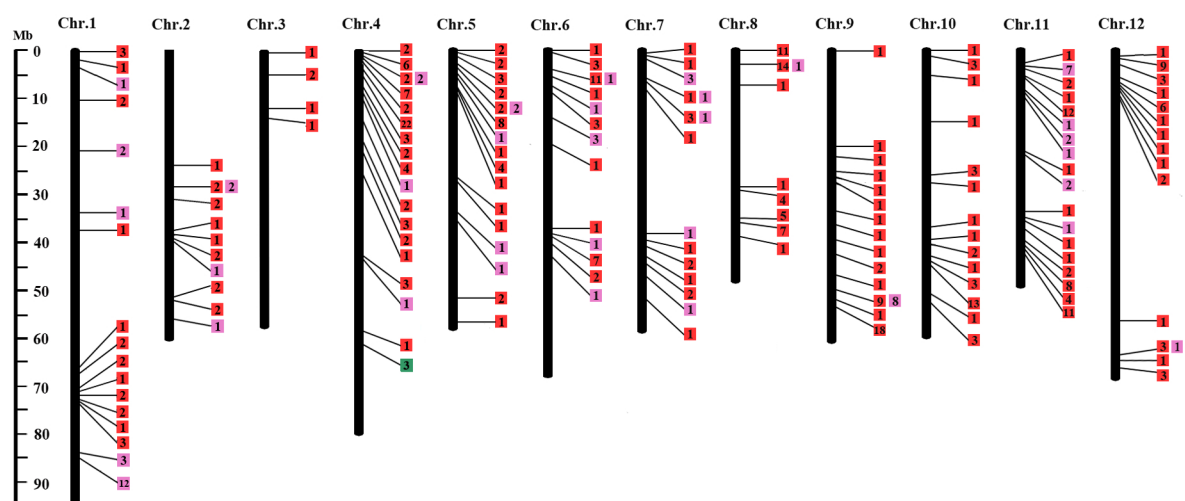**C**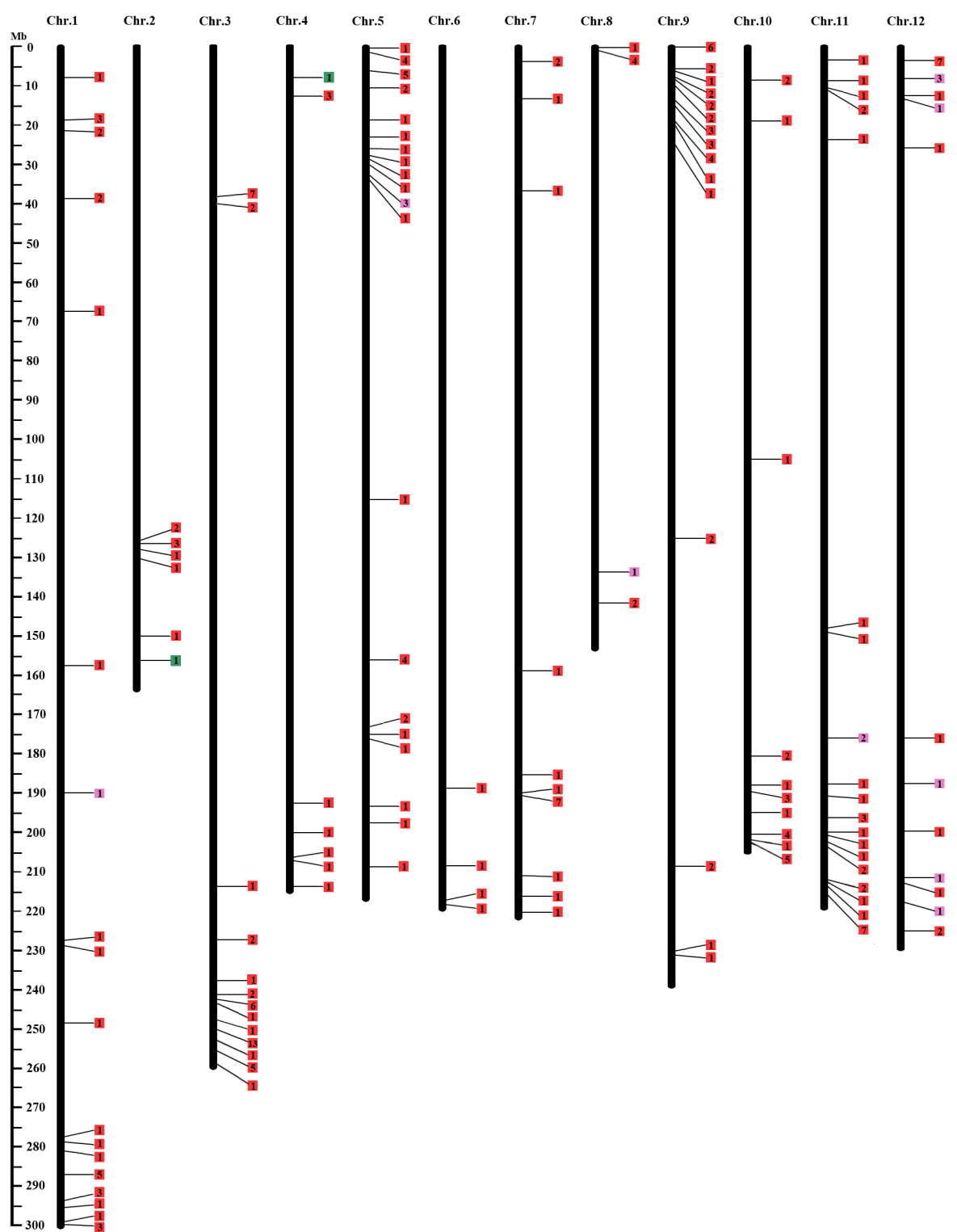

Supplement: Supplementary file 1 [file 1577FigureS1.pdf]

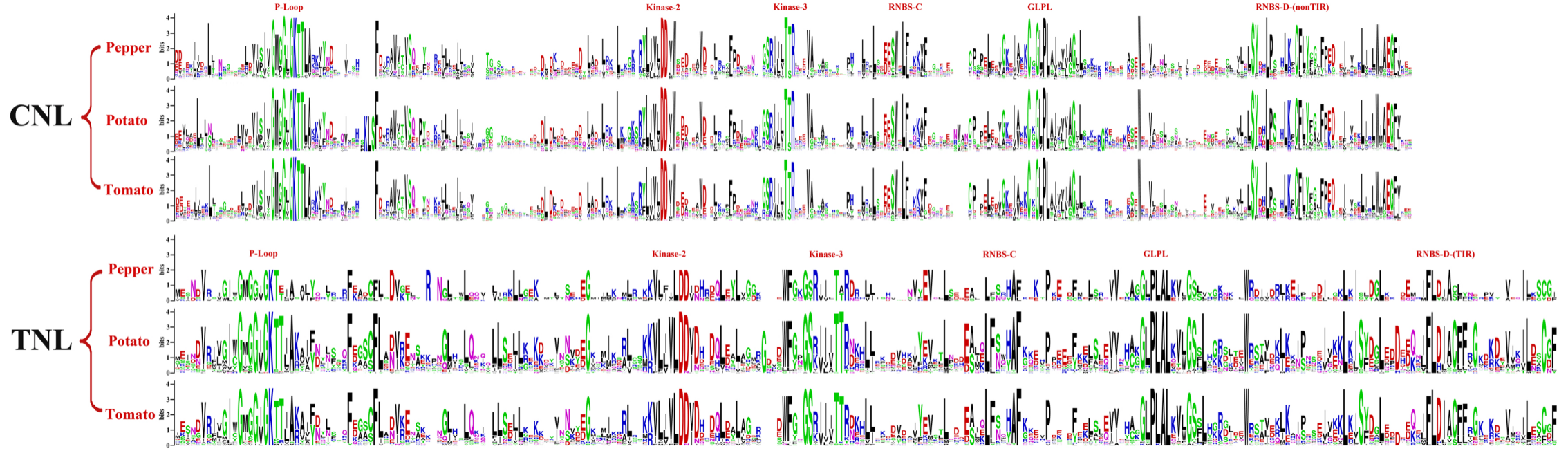

Supplement: Supplementary file 2 [file 1577FigureS2.pdf]

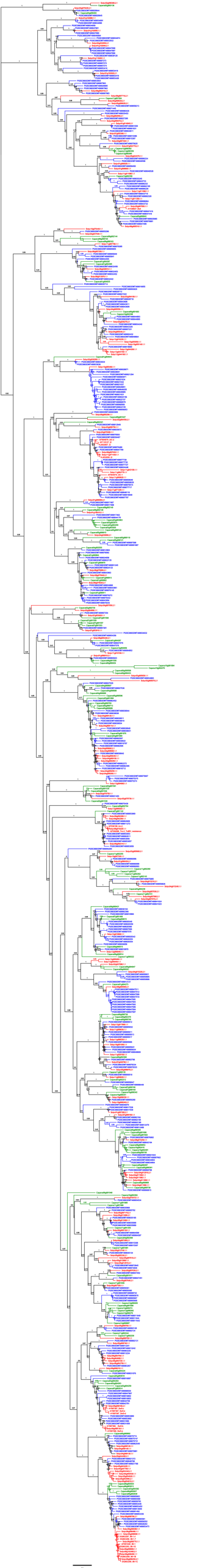

Supplement: Supplementary file 3 [file 1577FigureS3.pdf]

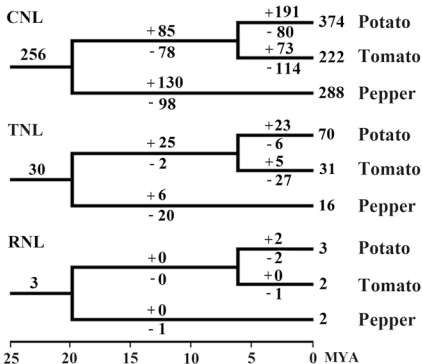

Supplement: Supplementary file 7 [file 1577FigureS7.pdf]
