# Supplementary material for: Distinct Patterns of Gene Gain and Loss: Diverse Evolutionary Modes of NBS-Encoding Genes in Three Solanaceae Crop Species
Source: G3 (Bethesda). 2017 Mar 28;7(5):1577–85. doi: 10.1534/g3.117.040485 (PMC5427506; doi:10.1534/g3.117.040485)
Supplement: Supplementary file 6 [file 1577FigureS6.pdf]

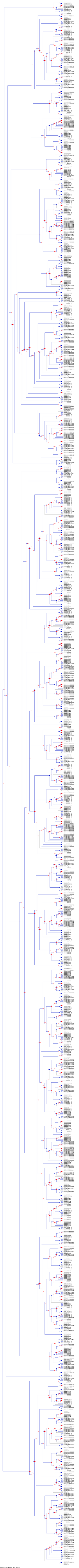

Image generated with Notung 2.6, on 2016-11-28
